# Generation of SARS-CoV-2 reporter replicon for high-throughput antiviral screening and testing

**DOI:** 10.1073/pnas.2025866118

**Published:** 2021-03-25

**Authors:** Xi He, Shuo Quan, Min Xu, Silveria Rodriguez, Shih Lin Goh, Jiajie Wei, Arthur Fridman, Kenneth A. Koeplinger, Steve S. Carroll, Jay A. Grobler, Amy S. Espeseth, David B. Olsen, Daria J. Hazuda, Dai Wang

**Affiliations:** ^a^Infectious Disease and Vaccines, Merck and Company, Inc., Kenilworth, NJ 07033

**Keywords:** SARS-CoV-2, COVID-19, replicon, antivirals, high-throughput antiviral screening

## Abstract

Severe acute respiratory syndrome coronavirus 2 (SARS-CoV-2) research and antiviral discovery are hampered by the lack of a cell-based virus replication system that can be readily adopted without biosafety level 3 (BSL-3) restrictions. Here, the construction of a noninfectious SARS-CoV-2 reporter replicon and its application in deciphering viral replication mechanisms and evaluating SARS-CoV-2 inhibitors are presented. The replicon genome is replication competent but does not produce progeny virions. Its replication can be inhibited by RdRp mutations or by known SARS-CoV-2 antiviral compounds. Using this system, a high-throughput antiviral assay has also been developed. Significant differences in potencies of several SARS-CoV-2 inhibitors in different cell lines were observed, which highlight the challenges of discovering antivirals capable of inhibiting viral replication in vivo and the importance of testing compounds in multiple cell culture models. The generation of a SARS-CoV-2 replicon provides a powerful platform to expand the global research effort to combat COVID-19.

## Significance

The urgency of curbing the COVID-19 pandemic has motivated many investigators to pivot their research to understand the basic biology of SARS-CoV-2 and to search for new pharmaceutical compounds for potential COVID-19 treatment. However, most SARS-CoV-2 studies require biosafety level 3 facilities, which are in high demand, costly, and difficult to access. To overcome these limitations, we engineered a SARS-CoV-2 replicon, which is a modified virus subgenome capable of self-replicating without producing infectious virus, allowing the viral replication to be studied in a conventional biomedical laboratory setting. The replicon system also provides a valuable tool to screen and test antiviral compounds in biologically relevant cells. Successful implementation of the technology will accelerate the development of effective treatment for SARS-CoV-2 infection.

The current pandemic of coronavirus disease 2019 (COVID-19) caused by the newly emerged coronavirus, severe acute respiratory syndrome coronavirus 2 (SARS-CoV-2), has led to more than 100 million infections and 2.44 million deaths as of February 18, 2021 (1). In response to this public health emergency, an unprecedented swift and concerted effort has been launched globally to identify safe and effective therapeutics and vaccines against the rapidly spreading virus. Presently, about 2,000 SARS-CoV-2 investigational programs and clinical trials have been registered (2). However, most of the treatment trials to date have focused on repurposing a limited number of existing antivirals approved for other indications, such as lopinavir/ritonavir, approved for treatment of HIV infection, or remdesivir, originally developed for treatment of Ebolavirus infection. Although compounds directly targeting SARS-CoV-2 are urgently needed, discovery and development of such compounds typically require a decade of dedicated effort. One of the major bottlenecks is that the replication of SARS-CoV-2 is not yet fully understood, and assays used to study viral genome replication and test SARS-CoV-2 compounds must be performed in biosafety level 3 (BSL-3) laboratories, which are not readily available to most researchers.

To address the need for a broadly accessible and robust cell-based SARS-CoV-2 replication platform, a SARS-CoV-2 replicon was constructed. In this replicon, the spike (S) protein open reading frame (ORF) was deleted and replaced with a gene encoding a firefly luciferase (Luc) and green fluorescence (GFP) fusion protein. The sequences of envelope (E), membrane (M), and their intergenic region were also deleted and replaced with a neomycin-resistance gene (Neo). The replicon maintained all the nonstructural protein genes and genetic elements necessary for replicating the full-length and subgenomic (sg) RNAs. The nonstructural proteins are encoded by two overlapping ORFs, ORF1a and ORF1b. ORF1a produces a polyprotein pp1a, while the −1 programed ribosomal frameshifting following the translation of ORF1a leads to production of an even larger polyprotein pp1a/b. The polyproteins are proteolytically cleaved to yield 16 nonstructural proteins, NSP1–16. The NSPs are responsible for viral replication and transcription, suppression of host innate immunity, and modulating other cellular functions. As a result, the replicon system can be used to evaluate antiviral activities of compounds targeting NSPs and to dissect the mechanisms of virus replication and pathogenesis. Deletion of the structural genes rendered the replicon defective in producing progeny virions, thereby enabling safe handling under BSL-2 conditions.

Another advantage of the transient replicon system over traditional live virus-based models is that the replicon RNA can be introduced to a variety of cell lines, allowing for assessment of viral replication under more physiologically relevant conditions. SARS-CoV-2 preferentially targets the respiratory tract but may also spread to other organs and tissues such as heart, liver, brain, and kidneys (3). In infected tissues, the virus has been detected in multiple cell types (3, 4). However, in tissue culture, very few cell lines are permissive to productive SARS-CoV-2 infection (5). As a result, most SARS-CoV-2 replication analysis and compound testing so far have been performed in Vero E6 cells, which may not fully recapitulate the complexity of infections in vivo.

In addition, a high-throughput antiviral assay has been developed using the described replicon system. Under those assay conditions, the sensitivities of viral replication to SARS-CoV-2 inhibitors were found to be cell line dependent, suggesting antiviral compounds may have different potencies against the virus infecting different cell types in vivo. Therefore, the replicon system also provides a valuable tool to screen, test, and optimize antiviral compounds and to elucidate their mechanism of action.

## Results

### Construction and Characterization of SARS-CoV-2 Replicon.

**Fig. 1.**
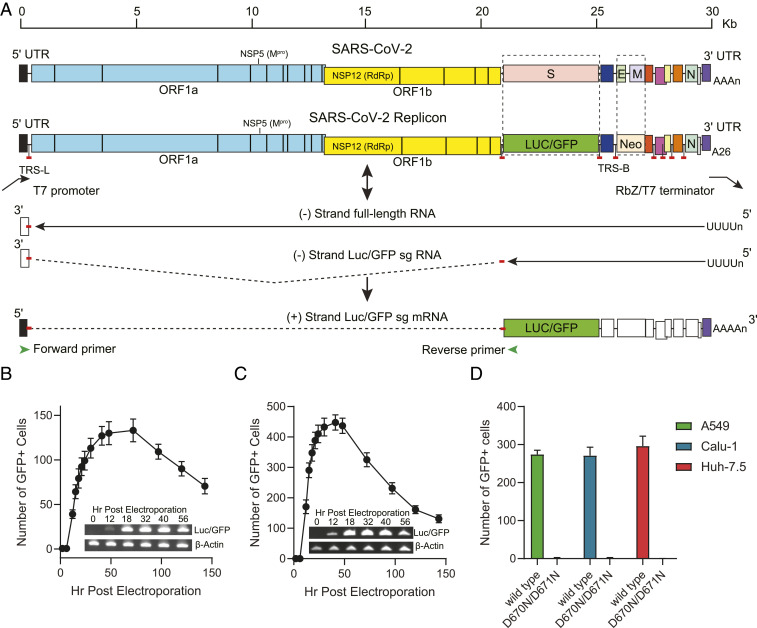
Construction and transcriptional analysis of SARS-CoV-2 replicon. (*A*) Schematic diagram of reporter replicon genome structure, replication, and sg mRNA production. The S and E/M ORFs in the wt viral genome were deleted and replaced with Luc/GFP and Neo genes, respectively. The full-length replicon cDNA was flanked by a T7 promoter and a polyA/HDV ribozyme and T7 terminator cassette. The in vitro-transcribed replicon RNA is copied by replicase complex to produce genomic or subgenomic-sized negative stranded RNAs (solid lines). The negative stranded sg RNAs are used as templates to produce the sg mRNAs for expression of reporter and structural proteins. The sg mRNAs consist of the leader at 5′-UTR of the genome and mRNA body sequences joined by a short and conserved sequence motif, the transcription-regulating sequence (TRS). The location of the sequences encoding major antiviral targets NSP5 (M^pro^) and NSP12 (RdRp), the untranscribed regions (dashed lines), TRS leader (TRS-L) and TRS body (TRS-B) are also indicated. (*B* and *C*) Kinetics of GFP expression post-electroporation and detection of the sg mRNA expressing Luc/GFP reporter products. 293T (*B*) and A549 (*C*) cells were electroporated with in vitro-transcribed replicon RNA, and the number of GFP-positive cells in each well containing 1 × 10^4^ cells were counted at indicated time points. Total RNA was also collected and purified at indicated times from electroporated cells. RT-PCR products from mRNAs expressing Luc/GFP or β-actin were confirmed by gel electrophoresis. (*D*) GFP reporter signals produced by the wt and RdRp mutant replicons. The A549, Calu-1, and Huh-7.5 cells were electroporated with T7 polymerase transcribed wt replicon RNA or the RdRp D760N/D761N double-mutant replicon RNA and transferred to 384-well plates. The number of GFP-positive cells in each well were counted at 30 h post-electroporation.

A bacterial artificial chromosome (BAC) system was employed to construct the SARS-CoV-2 replicon. As the full-length SARS-CoV-2 replicon is ∼28,000 nt, its cDNA, together with the flanking regions, was synthesized as five fragments with overlapping ∼30 base pairs and subsequently assembled into a BAC vector. The replicon was also linked to a T7 minimal promoter upstream of the 5′ untranslated region (UTR), and a cassette containing a 26-nt poly(A), a hepatitis D virus ribozyme (Rbz), and a T7 terminator at the 3′ end to yield precise viral genome terminus (Fig. 1*A*).

To produce the replicon RNA, the BAC containing the replicon cDNA was linearized by restriction enzyme digestion and then transcribed in vitro by T7 RNA polymerase as described in *Materials and Methods*. The 5′ end of the synthesized RNA was capped by an anti-reverse cap analog (ARCA). The replicon RNA was electroporated into indicated cell lines, and amplification of the replicon RNA was quantified by the expression of GFP or luciferase reporter genes. A similar in vitro transcription strategy has also been successfully used in the coronavirus reverse genetics systems to rescue recombinant SARS-CoV-2 viruses (6–8).

The replication of in vitro-transcribed RNA was initially examined in 293T cells. According to the coronavirus replication model (9), the viral genome is copied by a replication–transcription complex, both continuously to produce minus-strand templates for genome RNA synthesis and discontinuously to produce minus-strand templates for sg mRNA synthesis. These two types of RNAs have also been reported recently in the SARS-CoV-2–infected cells (10). The structural proteins such as S, E, M, and N are encoded by sg mRNAs (Fig. 1*A*). Therefore, GFP in the replicon RNA transfected cells is also likely expressed by the sg mRNAs. To confirm the synthesis of the Luc/GFP sg mRNAs, RT-PCR analysis was performed on the transcripts in the electroporated cells. The forward primer was positioned in the leader sequence of 5′-UTR, and the reverse primer was situated in the Luc/GFP coding region (Fig. 1*A*). This resulted in a primer pair only amplifying Luc/GFP sg mRNA. Consistent with the GFP expression, the Luc/GFP sg mRNA was detected as early as 12 h post-electroporation for both 293T and A549 cells (Fig. 1 *B* and *C*). The presence of the Luc/GFP sg mRNA also indicated that the in vitro-transcribed replicon RNA produces a functional replicase complex. The kinetics of GFP expression was then investigated at 0 to 148 h post-electroporation in 293T and A549 cells (Fig. 1 *B* and *C*). Consistent with findings in the RT-PCR analysis, GFP signals were observed at 12 h post-electroporation and reached the peak levels at 48 to 56 h post-electroporation for both cell lines. The number of GFP-positive cells declined more rapidly in A549 cells after 48 h post-electroporation, suggesting the replicon is less stable in those cells in comparison to 293T cells.

**Fig. 2.**
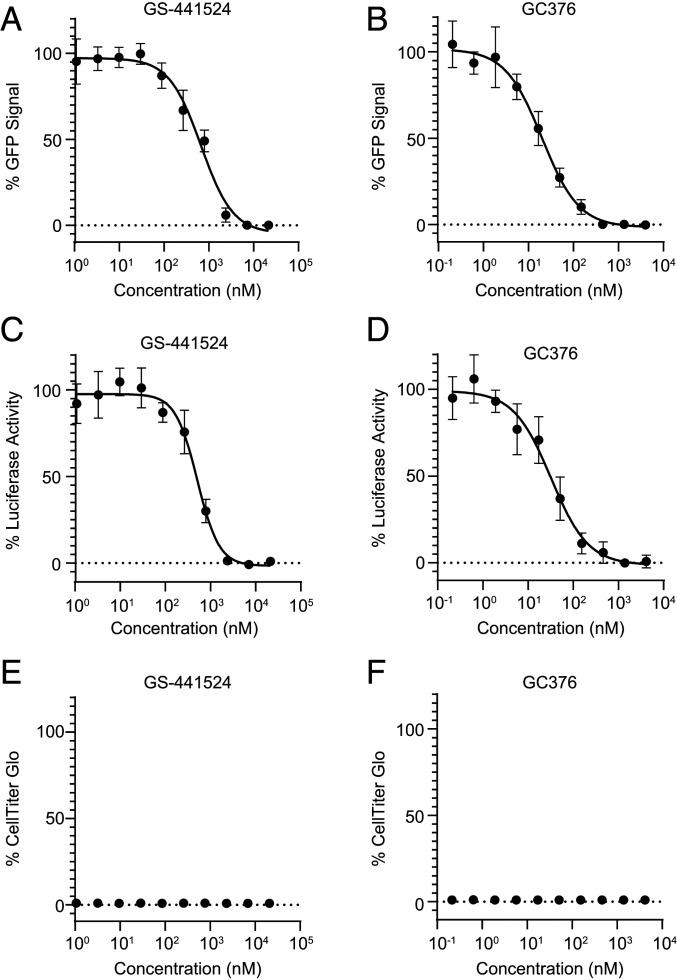
Dose-dependent responses of SARS-CoV-2 replicon reporter activity to GS-441524 and GC376. The 293T cells were electroporated with T7 polymerase transcribed replicon RNA and incubated with GS-441524 or GC376 compounds at indicated concentrations. The activities were determined by percentage of GFP or luciferase signals comparing to DMSO-treated cells (*A–D*). The cytotoxicity of GS-441524 and GC376 to the cells were also examined (*E* and *F*).

**Fig. 3.**
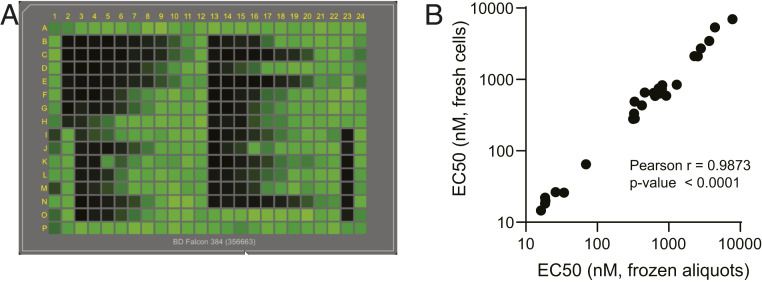
Adaptation of the SARS-CoV-2 replicon-based assay to high-throughput platform. (*A*) The plate view of GFP expression in replicon electroporated cells treated with compounds. The 293T cells were electroporated with T7 polymerase transcribed replicon RNA and incubated with control compounds in 384-well plates for 30 h. The plates were scanned using an Acumen eX3. (*B*) Correlation between the EC_50_ values measured using freshly or frozen electroporated cells. A total of 27 control compounds was tested using either fresh or cryopreserved 293T cells electroporated by SARS-CoV-2 replicon RNA as described in *Materials and Methods*. The strength of correlation is determined by the Pearson test.

In addition to 293T and A549 cells, other cell lines such as Calu-1 and Huh-7.5 cells were also demonstrated to support the replication of the SARS-CoV-2 replicon RNA, as evidenced by the GFP reporter gene expression in those cells following electroporation with the replicon RNA (Fig. 1*D*). Electroporation efficiencies ranged from ∼1.3% in 293T cells (Fig. 1*B*) to 3 to 4% in A549, Calu-1, and Huh-7.5 cells (Fig. 1 *C* and *D*). To further investigate whether the reporter signals are generated by de novo synthesized RNAs, two point mutations, D760N and D761N, were introduced to NSP12, which encodes RNA-dependent RNA polymerase (RdRp). The combination of the two mutations inactivates the RdRp and leads to a complete loss of native ribonucleotide incorporation (11). As expected, the mutant replicon RNA failed to express GFP in transfected A549, Calu-1, and Huh-7.5 cells (Fig. 1*D*). The data confirmed that reporter activities are a result of active replicon RNA replication and can be used as a readout to evaluate the effectiveness of potential inhibitors of enzymes required for replication of SARS-CoV-2.

### Inhibition of Replicon Replication by Antiviral Compounds.

In order to demonstrate the sensitivity of the SARS-CoV-2 replicon to coronavirus inhibitors with different mechanisms of action (MOA), a series of pilot experiments were performed in 293T cells. The compounds chosen for these initial studies were GS-441524 and GC376. GS-441524 is the parent compound of remdesivir, a prodrug originally discovered as an antiviral against Ebola virus (12), specifically targeting the RdRp (11). Remdesivir has been approved by the US Food and Drug Administration to treat hospitalized COVID-19 patients (13). Another notable compound, GC376, a broad-spectrum inhibitor against 3C or 3C-like proteases that has previously been used to treat feline infectious peritonitis caused by feline coronavirus (14), is also reported to exert potent inhibition for the main protease (M^pro^ or 3CL^pro^) encoded by NP5 gene of SARS-CoV-2 (15).

The 293T cells were electroporated with in vitro-transcribed and capped full-length replicon RNA, and then incubated with increasing concentrations of GS-441524 or GC376 in a 384-well plate. Inhibition of replication was determined by reduction of the number of GFP-positive cells or luciferase activities relative to DMSO control treated cells at 30 h postelectroporation. As shown in Fig. 2 *A*–*D*, both compounds exhibited antiviral activities in a dose-dependent manner, using either GFP or luciferase as a readout. The potencies of GS-441524 in inhibiting the SARS-CoV-2 replicon replication, determined by either percent GFP signal or percent luciferase activity, were 645 or 503 nM, respectively. The potencies of GC376 in inhibiting the SARS-CoV-2 replicon replication, determined by either percent GFP signal or percent luciferase activity, were 22.0 or 29.2 nM, respectively. The EC_50_ values measured by both reporters were comparable in these assays with the advantages of single-cell–based GFP readouts for continuous monitoring of reporter gene expression, and of population-based luciferase readouts for fast assay development. The luciferase assay also confirmed that GFP expression reduction resulted from inhibition of replicon replication, not from quenching of GFP fluorescence. No cytotoxicity was detected for either compound in the range of concentrations tested (Fig. 2 *E* and *F*).

### Antiviral Evaluation Using SARS-CoV-2 Replicon in Different Cell Lines.

Next, we sought to determine the impact of cell lines on the potencies of a panel of SARS-CoV-2 compounds measured by the replicon assay. Those compounds have previously been shown to block SARS-CoV-2 replication at different stages of its life cycle.

As described above, all cell lines tested including 293T, Vero, Huh-7.5, Calu-1, and A549 could support replication of SARS-CoV-2 replicon. The compounds selected in this study were viral entry inhibitors, nafamostat and camostat (16) targeting transmembrane serine protease 2 (TMPRSS2), endosome trafficking inhibitors, relacatib (17), and apilimod (17) targeting cathepsin L and PIKfyve, respectively, polymerase inhibitors, remdesivir (11) and its parent compound GS-441524, and the protease inhibitors GC373 (14, 18), GC376 (15), PF-00835231 (19), and Boceprevir (20, 21).

Since the SARS-CoV-2 replicon RNA was introduced to the cells by electroporation, bypassing the natural entry pathways of the virus, as expected, the SARS-CoV-2 entry inhibitors were inactive in the replicon-based assay (Table 1). Also, due to the deletion of the structural proteins, S, E, and M, this replicon assay is unlikely to be capable of identifying compounds that inhibit SARS-CoV-2 assembly or release.

**Table 1.** Antiviral activity against SARS-CoV-2 replicon

Target	Compound	MOA	Cells	EC_50_, nM
TMPRSS2	Nafamostat	Entry	293T	>42,000
			Vero E6	>42,000
			Calu-1	>42,000
			A549	>42,000
	Camostat	Entry	293T	>42,000
			Vero	>42,000
			Calu-1	>42,000
			A549	>42,000
PIKfyve	Apilimod	Endosome entry	Calu-1	>42,000
			Huh-7.5	>42,000
Cathepsin L	Relacatib	Endosome entry	293T	>42,000
			Vero	>42,000
			Calu-1	>42,000
			A549	>42,000
RdRp	GS-5734	RNA synthesis	293T	15.6 ± 5.1
	(Remdesivir)	(nucleotide prodrug)	Vero	2,240
			Huh-7.5	18.9
			Calu-1	31.5 ± 23
			A549	184
	GS-441524	RNA synthesis	293T	603 ± 131
	(Remdesivir parent)	(nucleoside)	Vero	302 ± 20
			Huh-7.5	1,030 ± 354
			Calu-1	1,330 ± 270
			A549	1,468 ± 163
Mpro	GC373	Polyprotein cleavage	293T	20.3 ± 5.3
			Vero	309 ± 43
			Calu-1	30.1 ± 5.8
			A549	539 ± 69
	GC376	Polyprotein cleavage	293T	20.8 ± 5.4
			Vero	237 ± 74
			Huh-7.5	19.4 ± 4.9
			Calu-1	19.8 ± 2.0
			A549	570 ± 182
	PF-00835231	Polyprotein cleavage	293T	75.2 ± 19.1
			Vero	2,190 ± 221
			Huh-7.5	175 ± 56
			Calu-1	24.7 ± 3.8
			A549	76.3 ± 16.2
	MK-3034	Polyprotein cleavage	293T	3,550 ± 347
	(Boceprevir)		Vero	16,600 ± 4,480
			Calu-1	4,790 ± 405
			A549	19,900 ± 517

Remdesivir and GS-441524 demonstrated antiviral effects in all cell lines, albeit to various degrees. The highest potency of remdesivir was found in 293T cells, with an EC_50_ value of 15.6 nM. In contrast, a much higher concentration of the compound is needed to achieve 50% inhibition in Vero cells (EC_50_ of 2,240 nM), which could be attributed to inefficient cellular uptake and/or conversion of the nucleoside to the active triphosphate form. The reported EC_50_ values of remdesivir against wild-type (wt) SARS-CoV-2 infection were 442 nM in ACE2 stably transfected A549 cells (22), and 770 to 1,650 nM in Vero E6 cells (23, 24), consistent with the potencies of remdesivir measured in this study (Table 1). GS-441524 was relatively weaker in inhibiting the replicon activity in most cell lines compared to remdesivir.

The M^pro^ inhibitors, GC376, PF-00835231, and Boceprevir, also exhibited notable antiviral activities in different cell lines using the replicon-based assay. The sensitivity of the replicon to these compounds was nevertheless cell line dependent. GC376 and its parent compound GC373 (14, 18) were highly potent in 293T and Calu-1 cells, but only modestly active in A549 and Vero cells. In contrast, the EC_50_ value of PF-00835231 ranged from 32 to 175 nM in Calu-1, A549, and Huh-7.5 cells, and was 2,190 nM in Vero cells. Boceprevir was the least potent among the M^pro^ compounds tested in this study.

Previously, it has been reported that Vero cells express high levels of the efflux transporter P-glycoprotein (p-gp) encoded by MDR1 or ABCB1 (25), which efficiently exports PF-00835231 and reduces the intracellular concentration of the compound (19). To determine the effect of p-gp on the activities of other M^pro^ inhibitors in Vero cells and the role of p-gp in other cell culture systems, the antiviral assay was then performed in the presence of p-gp inhibitors CP-100356 and elacridar, respectively. No antiviral activities were observed for either CP-100356 or elacridar at concentrations up to 2 µM when tested alone. Consistent with previous report (19), p-gp inhibitors dramatically increased the potency of PF-00835231 in Vero cells. There was also a slight increase of the activities of GC373, GC376, and Boceprevir in these cells. In contrast, the p-gp inhibitors did not cause any significant shift in the EC_50_ values of those compounds in A549 or Calu-1 cells.

### Adaptation of SARS-CoV-2 Replicon-Based Assay for High-Throughput Compound Screening.

The current replicon assay is performed in 384-well plates using automated liquid handling systems (Fig. 3*A*); therefore, the format is capable of screening large compound libraries. Results are available as early as 24 h following addition of drug. When conducting the high-throughput screening of compounds, multiple factors may impact the assay performance or variability. Those contributing factors include bacmid integrity, RNA quality, and overall cell health at the time of experiment. To further improve the assay consistency, a system using cryopreserved cell aliquots to screen and test compounds was developed. The EC_50_ values of the compounds determined using frozen cell aliquots were within error of the assay with those determined using freshly electroporated cells (Fig. 3*B*).

## Discussion

Over the past decades, due in large part to the advances in reverse genetics, replicon systems have been established for a number of RNA viruses, such as hepatitis C virus, SARS-CoV-1, Middle East respiratory syndrome coronavirus, dengue virus, West Nile virus, Zika virus, poliovirus enterovirus 71, hepatitis E virus, chikungunya virus, and many others (26). For highly virulent viruses, the replicon assay system provides a safe and high-throughput method to screen and evaluate compounds. The replicon-based assay also allows the identification of molecules that target proteins other than RdRp and M^pro^ whose functions are unknown or for which in vitro enzymatic assays are not yet understood, and elucidation of the compound mechanism of action via resistant selection (27, 28). Moreover, replicons make it possible to identify inhibitors against viruses that proved difficult to propagate in tissue culture, such as hepatitis C virus (29, 30) and norovirus. Replicons have and will continue to play a crucial role in the discovery of new drugs against viral pathogens.

The large size of the coronavirus genomes and the instability of certain cDNA sequences in *Escherichia coli* were the major obstacles in engineering the SARS-CoV-2 replicons. Multiple strategies, such as using BAC or vaccinia virus vectors to propagate and modify their cDNA genomes, have been implemented to construct replicons for human coronavirus 229E (31) and SARS-CoV-1 (32). During the preparation of this manuscript, a SARS-CoV-2 replicon generated by in vitro ligation of cDNA fragments has also been reported (33). The design of those replicons also differed slightly in the structure gene deletions and the reporter genes used. Even though in vitro ligation offers a rapid approach in generating SARS-CoV-2 replicon as described by Xia et al. (33), the BAC vector allows more consistent and scalable replicon RNA production to accommodate downstream high-throughput antiviral assays, which is key for successful adoption of the system worldwide. Two of the most commonly used bioluminescence and fluorescence proteins, GFP and firefly luciferase, were chosen as reporters to further facilitate the standardization of the system.

Besides the current replicon design, a cytomegalovirus (CMV) immediate-early gene promoter was evaluated to initiate the transcription of SARS-CoV-2 replicon, an approach reported previously for generation of SARS-CoV-1 replicon (32). The replicon cDNA under the control of a CMV promoter can be directly transfected into cells without the need of in vitro transcription. However, the replicon cDNA itself produced background GFP and luciferase signals, and the reporter activities were more variable from experiment to experiment.

We have attempted to establish a stable cell line that could maintain the replicon RNAs but to date have been unsuccessful. SARS-CoV-2 replicon might be toxic to the cells or cleared by intracellular innate immunity. Replicon toxicity has also been observed by Xia et al. (33). Attenuation of replicon replication therefore might be needed for the cells to tolerate the virulence of SARS-CoV-2 proteins. One approach to achieve this is to engineer TRS-L and TRS-B sequences that are not found naturally in CoV into this replicon as described by Graham et al. (34). In addition, rewiring the transcription regulatory networks would further enhance the biosafety of the replicon system by suppressing any potential for generation of a novel CoV as a result of accidental infection of cells expressing the SARS-CoV-2 replicon with another CoV. Efforts to suppress or modify innate immunity to permit the tolerance of SARS-CoV-2 replicon are also ongoing.

The utility of the replicon system for studying SARS-CoV-2 genome replication and gene expression was demonstrated by the observations that the replicon is fully capable of producing sg mRNAs as seen in virus-infected cells (Fig. 1). The expression of reporter genes could be inhibited by point mutations in RdRp or antiviral treatment. There were dramatic differences in potencies of the SARS-CoV-2 protease inhibitors in different cell lines. Multiple factors may contribute to the cell line-dependent compound sensitivity. It is conceivable that the replicon may have different replication kinetics in different cells. High level expression of the protease may reduce the relative effectiveness of the compounds. The shift of potency is unlikely caused by the differences in electroporation efficiencies, since the A549, Calu-1, and Huh-7.5 showed similar number of cells expressing GFP in the assay (Fig. 1*D*). Another possible explanation is that rates of compound uptake, efflux, and metabolism may be different in those cells, as shown in Vero cells (Table 2). Understanding the impact of cell lines on SARS-CoV-2 response to those compounds may also have an important implication in the clinical development of the drugs.

**Table 2.** Effect of p-gp inhibitors on the potency of compounds against SARS-CoV-2 replicon

	EC_50_, nM		
Compound	Vero	A549	Calu-1
GC373	309 ± 43	539 ± 69	30.1 ± 5.8
GC373 + 0.5 µM CP-100356	99.2 ± 9.7	581 ± 16	29.4 ± 4.7
GC373 + 2 µM CP-100356	64.2 ± 9.9	403 ± 30	20.9 ± 3.9
GC373 + 0.5 µM Elacridar	54.2 ± 0.4	656 ± 98	33.7 ± 8.4
GC373 + 2 µM Elacridar	35.8 ± 4.1	510 ± 147	27.0 ± 5.7
GC376	237 ± 74	570 ± 182	19.8 ± 2.0
GC376 + 0.5 µM CP-100356	63.8 ± 2.9	551 ± 3	9.16 ± 2.70
GC376 + 2 µM CP-100356	44.0 ± 2.9	481 ± 83	9.42 ± 1.05
GC376 + 0.5 µM Elacridar	93.3 ± 15.7	631 ± 94	10.3 ± 2.04
GC376 + 2 µM Elacridar	46.4 ± 10.7	651 ± 124	9.40 ± 1.94
PF-00835231	2,190 ± 221	76.3 ± 16.2	24.7 ± 3.8
PF-00835231+ 0.5 µM CP-100356	68.4 ± 5.7	61.0 ± 4.0	9.67 ± 1.10
PF-00835231+ 2 µM CP-100356	17.3 ± 0.6	33.6 ± 2.3	9.59 ± 1.73
PF-00835231+ 0.5 µM Elacridar	25.0 ± 6.1	61.5 ± 17.2	12.4 ± 1.4
PF-00835231+ 2 µM Elacridar	16.3 ± 2.0	50.3 ± 9.3	9.68 ± 2.0
Boceprevir	16,600 ± 4,480	19,900 ± 517	4,790 ± 405
Boceprevir + 0.5 µM CP-100356	7,800 ± 1,930	19,800 ± 1480	4,340 ± 1,246
Boceprevir + 2 µM CP-100356	4,300 ± 1,170	17,900 ± 892	4,050 ± 857
Boceprevir + 0.5 µM Elacridar	4,000 ± 1,290	16,500 ± 699	4,540 ± 570
Boceprevir + 2 µM Elacridar	4,290 ± 1,230	16,800 ± 261	4,080 ± 553
CP-100356	>4,670	>4,670	>5,000
Elacridar	>4,670	>4,670	>8,400

## Materials and Methods

### Experimental Design.

The objective of this study is to develop a noninfectious, high-throughput and cell line-independent SARS-CoV-2 replicon-based antiviral screening and testing system. The full-length cDNA of a SARS-CoV-2 reporter replicon was first synthesized and assembled into a BAC. Replicon RNA is produced in vitro using T7 polymerase and introduced to cells through electroporation. The amplification of the replicon RNA is then confirmed by the expression of sub genomic RNAs, and by the loss of the reporter activities as a result of RdRp inactivation. The replicon was further evaluated in an antiviral assay using known SARS-CoV-2 inhibitors. The impact of cell lines and efflux transporter on antiviral potencies of the compounds was also determined. The replicon-based assay has been automated and adapted to a 384-well plate format for high-throughput compound library screening. The detailed methods on the replicon construction and antiviral assays are described in *SI Appendix*.

### Cell Lines.

Human embryonic kidney 293T (Clontech; 632180), human lung carcinoma A549 (ATCC; CCL-185), and human hepatoma Huh7.5 (Apath) cells were maintained in high-glucose Dulbecco’s modified Eagle’s medium (DMEM) (Invitrogen; 10-013-CV); African green monkey kidney Vero (ATCC; CCL-81) and human lung epidermoid carcinoma Calu-1 (ATCC; HTB-54) cells were cultured in Eagle’s minimum essential medium (ATCC; 30-2003) and McCoy’s 5a medium Modified (ATCC; 30-2007), respectively. All of the media were supplemented with 10% fetal bovine serum (FBS) (Invitrogen; 10082147) and 1% penicillin/streptomycin. Cells were grown at 37 °C with 5% CO_2_.

### Replicon Construction.

The replicon sequence is derived from the SARS-CoV-2 isolate Wuhan-Hu-1 (GenBank: NC045512). The replicon cDNA is placed under a T7 (5′-TAA​TAC​GAC​TCA​CTA​TAG-3′) promoter. The T7 RNA polymerase initiates transcription at the underlined G in the promoter sequence. Five fragments spanning the T7 promoter, full-length SARS-CoV-2, polyA/RbZ/T7 terminator (Fig. 1*A*), named F1 to F5, with ∼30-bp overlap were synthesized by Genewiz. pSMART BAC vector (Lucigen) was digested with NotI. F1 and F5 fragments were digested with MluI. Equimolar amounts of linearized pSMART BAC vector, F1, and F5 were ligated using Gibson Assembly kit (NEB) according to manufacturer’s instruction, resulting in pSMART BAC F(1,5). Equimolar amounts of pSMART BAC F(1,5) digested with AatII and AscI, and F2 and F4 digested with MluI, were ligated using Gibson Assembly kit, resulting in pSMART BAC F(1,2,4,5). Finally, pSMART BAC F(1,2,4,5) digested with AatII and AscI, and F3 digested with SwaI were ligated together using Gibson Assembly Kit, resulting in the full-length noninfectious SARS-Cov-2 replicon construct pSMART BAC-T7-scv2-replicon.

### RdRp Mutant Replicon Construction.

The RdRp mutations were made to the replicon by BAC recombineering (35). An ampicillin (Amp) cassette was introduced to the RdRp immediately downstream of D761 residue, followed by Gibson assembly approach to replace the Amp cassette with the D760N and D761N mutations. pSMART BAC-T7-scv2-replicon was transformed into SW102 *E. coli* host expressing the bacterial phage λ Red genes *gam*, *bet*, and *exo* which are controlled by *ts* repressor *c*I857. The Red enzymes are able to mediate homologous recombination between DNA fragments with overlapping sequences as short as 30 bp. The fragment containing an Amp cassette flanked by AscI restriction sites and adjacent homologous sequence was amplified by PCR with primers 5′-TTT​GTG​AAT​GAG​TTT​TAC​GCA​TAT​TTG​CGT​AAA​CAT​TTC​TCA​ATG​ATG​ATA​CTC​TCT*GGC​GCG​CC*GGA​ACC​CCT​ATT​TGT​TTA​TT-3′ and 5′-CAT​CTT​AAT​GAA​GTC​TGT​GAA​TTG​CAA​AGA​ACA​CAA​GCC​CCA​ACA​GCC​TGT​AAG​ACT*GGC​GCG​CC*TTA​CCA​ATG​CTT​AAT​CAG​TG-3′ using plasmid pcDNA3.1(+) as a template. The PCR fragment was then digested with Dpn I at 37 °C for 1 h and purified using the QIAquick PCR purification kit (Qiagen). To prepare the competent SW102 containing the pSMART BAC-T7-scv2-replicon, the *E. coli* host was first cultured in LB broth with 12.5 µg/mL chloramphenicol for 4 h to reach log phase at 32 °C, followed by heat shock treatment at 42 °C for 15 min to induce expression of λ Red genes. After three washes with ice-cold water, 100 ng of PCR fragment containing Amp cassette was electroporated into the competent cells. After 2 h of recovery at 32 °C, the electroporated cells were plated onto LB plates containing 100 µg/mL carbenicillin and 12.5 µg/mL chloramphenicol, and then cultured overnight at 32 °C. Correct clones were identified by colony PCR followed by restriction enzyme digestion. The pSMART BAC-T7-scv2-replicon-Amp DNA was prepared using NucleoBond BAC 100 kit (Takara; catalog #740579). A fragment containing the RdRp D760N D761N mutation sequence was amplified by PCR using primers 5′- CGT​AAA​CAT​TTC​TCA​ATG​ATG​ATA​CTC​TCT​AAC​AAT​GCT​GTT​GTG​TGT​TTC​AAT​AG-3′ and 5′- CAA​AGA​ACA​CAA​GCC​CCA​ACA​GCC​TGT​AAG​ACT​GTA​TGC​GGT​GTG​TAC​ATA​GC-3′. pSMART BAC-T7-scv2-replicon-AMP DNA was digested with AscI and then ligated to the fragment containing the RdRp D670N/D671N mutations using Gibson Assembly kit resulting in pSMARTBAC-T7-scv2m-replicon.

### RNA in Vitro Transcription and Electroporation.

The replicon vectors (pSMART-T7-scv2-replicon or pSMART-T7- scv2m-replicon) were first linearized by SwaI (NEB), and then purified by phenol/chloroform extraction and ethanol precipitation. The fragments were dissolved in nuclease-free water. The mMESSAGE mMACHINE T7 μLtra transcription kit (Invitrogen), which includes a cap analog ARCA, was used to generate the replicon RNAs in the correct orientation from the linearized vector according to manufacturer’s instruction. Briefly, 100 µL of T7 transcription reaction, containing 4 µg of linearized BAC and 15 µL of extra GTP, was incubated at 37 °C for 2.5 h to increase the length of the transcripts. After incubation, 5 µL of TURBO DNase was added and the reaction was incubated at 37 °C for 15 min to digest DNA. The resulting RNA was purified by Monarch RNA cleanup kit (NEB).

The cells were harvested using TrypLE Select (Thermo Fisher Scientific), washed three times with PBS, and resuspended in MaxCyte electroporation buffer to 1 × 10^8^ cells/mL. For 1 × 10^6^ of cells, 1 µg of replicon RNA was added into resuspended cells. The mixture was immediately transferred to a MaxCyte processing assembly (PA) (OC-100, OC-400, or R-1000), and electroporation was carried out by MaxCyte STX with the preloaded programs for transfecting different types of cells. After resting in PA for 20 min, transfected cells were transferred into 30 mL of complete DMEM (DMEM supplemented with 10% FBS and 1% penicillin–streptomycin). Following cell density adjustment (2 × 10^5^ cells per mL), the RNA-electroporated cells were plated into 384-well compound assay plates.

### Cryopreservation of Electroporated Cells.

The 293T cells were prepared and electroporated as described above. After resting for 20 min, the cells were pelleted, resuspended in freezing media (45% FBS, 45% complete DMEM, 10% DMSO) at 2 × 10^7^ cells per mL, and frozen in a Mr. Frosty Freezing container (Thermo Fisher Scientific) at −70 °C.

### Detection of sg mRNA Expressing Luc-GFP Fusion Protein.

Total RNAs were extracted from 1.2 × 10^6^ cells using RNeasy mini kit (Qiagen) at 0, 12, 18, 32, 40, and 56 h post-electroporation. cDNA was synthesized and amplified using a SuperScript IV One-Step qRT-PCR kit (Thermo Fisher Scientific) according to manufacturer’s instructions. The PCR products were analyzed on an E-Gel 1% agarose (Thermo Fisher Scientific). The sequences of Luc/GFP primer set used to amplify the sg mRNA were 5′-AGG​TTT​ATA​CCT​TCC​CAG​GT-3′ and 5′-TTT​GTA​TTC​AGC​CCA​TAG​CG-3′, and the sequences of β-actin primer set were 5′-GAG​CAC​AGA​GCC​TCG​CCT​TT-3′ and 5′-TGG​GGT​ACT​TCA​GGG​TGA​GG-3′.

### Compound Testing.

Compound plates were prepared by dispensing compounds (0.2 µL/well) dissolved in DMSO into wells of 384-well poly-d-lysine–coated plates (Corning; 356663) or cell culture-treated, flat-bottom plates (Corning; 3571) using an ECHO acoustic dispenser. Each compound was tested in a 10-point serial threefold dilution (final concentrations, 42,016 to 2.1 nM). Controls included DMSO only and GC-441524 (final concentration, 10 µM). A total of 10,000 transfected cells were added (50 µL/well) using Agilent Bravo to compound plates, and the cells were maintained at 37 °C/5% CO_2_/90% relative humidity. Reporter activities in the compound treated cells were quantified at 30 h posttransfection, by counting the number of green cells in each well using an Acumen eX3 scanner or by measuring luciferase activity with Steady-Glo Luciferase assay system (Promega) using EnVison (Perkin-Elmer).

### Cytotoxicity Assay.

Compound toxicities in replicon-transfected cells were determined via the CellTiter-Glo (CTG) luminescent assay (Promega), where the number of viable cells is determined based on the quantitation of ATP. After the compound plates were scanned in the Acumen eX3, they were equilibrated to room temperature for 30 min. The CTG assay was carried out according to manufacturer’s protocol. Briefly, 30 µL of the premixed CTG reagents was added into each well using the Agilent Bravo. After gently mixing, the reaction was incubated at room temperature for 10 min, and luminescent signals were measured using the EnVision (Perkin-Elmer).

### Quantification and Data Analysis.

All numerical data are presented as the mean ± SD. EC_50_ was determined by nonlinear four-parameter curve fitting using ActivityBase. Data presented in Fig. 1 were regraphed in Prism (GraphPad).

## Supplementary Material

Supplementary File

## Data Availability

All study data are included in the article and/or *SI Appendix*.
