# Supplementary material for: Generation of SARS-CoV-2 reporter replicon for high-throughput antiviral screening and testing
Source: Proc Natl Acad Sci U S A. 2021 Mar 25;118(15):e2025866118. doi: 10.1073/pnas.2025866118 (PMC8053989; doi:10.1073/pnas.2025866118)
Supplement: Supplementary File [file pnas.2025866118.sapp.pdf]

PNAS
www.pnas.org

Supplementary Information for

Generation of SARS-CoV-2 Reporter Replicon for High Throughput Antiviral Screening and Testing

Xi He, Shuo Quan, Min Xu, Silveria Rodriguez, Shih Lin Goh, Jiajie Wei, Arthur Fridman, Kenneth A. Koeplinger, Steve S. Carroll, Jay A. Grobler, Amy S. Espeseth, David B. Olsen, Daria J. Hazuda and Dai Wang

Correspondence: Dai Wang, Ph.D.
Email: dai_wang@merck.com

This PDF file includes:

Materials and methods for pSMART BAC-T7-scv2-replicon construction
Materials and methods for antiviral compound testing

pSMART BAC-T7-scv2-replicon Construction

Materials

pSMART BAC vector (Lucigen)
replicator v2.0 electrocompetent cells (Lucigen)
NEBuilder HiFi DNA Assembly Kit (NEB)
Restriction enzymes Mlu I, Swa I, Aat II, Asc I (NEB)
Gene Pulser (Bio-Rad)

5’ and 3’ end sequences of the synthetic cDNA fragments

Fragment		Sequences
F1	5’	CGCGTTTGACCATGTTGGTATGATTTAAATTCAGTGCGGCCGCTAATACGACTCACTATAGATTAAAGGTTTATACCTTCC
	3’	TACAAAATATCTAGTACAACAGGAGTCACCTGACGTCATCGGCGCGCCCCCGAGGCTACAAACGCTCTCATCGACAAGACGCGT
F2	5’	ACGCGTTACAAAATATCTAGTACAACAGGAGTCACCTTTTGTTATGATGTCAGCACCACCTGCTC
	3’	CTTATGACAGCAAGAACTGTGTATGATGATGACGTCATCGGCGCGCCTCCAGGGACCACCTGGTACTGGTAAGAGTCACGCGT
F3	5’	ATTTAAATCTTATGACAGCAAGAACTGTGTATGATGATGGTGCTAGGAGAGTGTGGAC
	3’	TATGCAAAAGTATTCTACACTCCAGGGACCACCTGGTACTGGTAAGAGTCATTTAAAT
F4	5’	ACGCGTTCCAGGGACCACCTGGTACTGGTAAGAGTCATTTTGCTATTGGCCTAGCT
	3’	TGAGCGGCTACGTTAACAACCCCGAGGCTACAAACGCTCTCATCGACAAGACGCGT
F5	5’	ACGCGTCCCGAGGCTACAAACGCTCTCATCGACAAGGACGGCTGGCTGCACAGCGG
	3’	TAGCTTCTTAGGAGAATGACAAAAAAAAAAAAAAAAAAAAAAAAAAGGGTCGGCATGGCATCTCCACCTCCTCGCGGTCCGACCTGGGCATCCGAAGGAGGACGCACGTCCACTCGGATGGCTAAGGGAGAGCCTGCAGTAGCATAACCCCTTGGGGCCTCTAAACGGGTCTTGAGGGGTTTTTTGGCGGCCGCTTCTATAGTGTCACCTAAATACTAGTGACTCCAGCACGCGT

Green: T7 promoter
Red: overlapping sequences
Blue: HDV ribozyme
Purple: T7 Terminator
Underline: restriction sites

Method

The replicon sequence is derived from the SARS-CoV-2 isolate Wuhan-Hu-1 (GenBank: NC045512). The replicon cDNA is placed under a T7 (5′ TAATACGACTCACTATAG 3′) promoter. The T7 RNA polymerase initiates transcription at the underlined G in the promoter sequence. Five fragments spanning the T7 promoter, SARS-CoV-2-EGFP-Luc, polyA/RbZ/T7 terminator (Fig. 1A), named F1 to F5, with 30-60 bps overlap containing homologous sequence and restriction enzyme sites (Table 2) were synthesized and cloned into a high-copy-number pUC57-kan plasmid by Genewiz (South Plainfield, NJ). pSMART BAC vector (Lucigen) was linearized by Not I digestion. F1 and F5 fragments were released from pUC57-Kan by Mlu I digestion. Equimolar amounts of linearized pSMART BAC vector, F1, and F5 were ligated together using NEBuilder HiFi DNA Assembly Kit (NEB) according to manufacturer’s instruction. 0.5 µl of reaction mixture were transformed into BAC-optimized replicator v2.0 electrocompetent cells (Lucigen) using Gene Pulser (Bio-Rad) with the condition of 1.5kV, 200 Ω, and 25 µF. pSMART BAC F(1,5) positive clones were selected by colony PCR. In the next step pSMART BAC F(1,5) was linearized with Aat II and Asc I, and F2 and F4 were cloned into pSMART BAC F(1,5) using the same method resulting in pSMART BAC F(1,2,4,5). Finally, F3 digested with Swa I was cloned into pSMART BAC F(1,2,4,5), resulting in the full-length non-infectious SARS-Cov-2 replicon construct pSMART BAC-T7-scv2-replicon.

Antiviral compound testing

Materials

DNA linearization and in vitro transcription:
Safe-lock microcentrifuge tubes 1.5mL: Eppendorf Cat# 226000028
SwaI: NEB R0604L
Phenol solution (pH8.0): Sigma-Aldrich Cat# P4557
Phenol/Chloroform/Isoamyl alcohol (25:24:1), pH6.7/8.0: Fisher BioReagents Cat# BP1752I-100
Chloroform/Isoamyl alcohol mixture: Sigma-Aldrich Cat# SIALMSD-25668-100ML
Ethanol: Decon Labs Cat#2716
70% ethanol: Fisher bioreagents Cat# BP8203
3M NaAc: Amresco Cat# E498-100ml
mMESSAGE mMACHINE T7 Ultra Transcription kit: Invitrogen Cat# AMB13455
Monarch RNA Cleanup Kit (500 ug): NEB Cat# T2050L

Cell maintenance media/ assay media:
DMEM (Dulbecco's Modified Eagle's Medium): Corning Cat# 10-013-CV
10% FBS (heat inactivated): Sigma Cat#F4135
100 u/ml Penicillin/streptomycin: Gibco Cat# 15140-122
Filtered

TrypLE Select (used to detach cells): Gibco Cat# 12563011

Cell Maintenance:
HEK 293T cells: Split and seed ~60,000 cells per mL every 3 to 4 days. Maintain cells in DMEM + 10% FBS+ 100 μ/mL pen/strep

Electroporation:
PBS pH7.4, Ca and Mg free: Gibco Cat# 10010-023 (or equivalent)
MaxCyte electroporation buffer: Cat# EPB1
Processing Assembly OC-400: Cat# SOC-4

Methods

DNA linearization

1. Digest replicon plasmid DNA (pSMART-T7-scv2-replicon or pSMART-T7- scv2m-replicon) with SwaI (NEB) according to the following conditions (note that the reaction can be scaled up or down accordingly):

	Volumes (µL)
Plasmid DNA (60 µg)	*x*
10X Buffer 3.1	30
SwaI (240 units at 10U/µL)	24
Nuclease-free water	(300-54-*x*)
Total digest volume	300

2. In a thermo cycler, digest the mix at 25°C for 2 hours, followed by inactivation at 65°C for 15min. If necessary, transfer the reaction mix into a microcentrifuge tube (labeled "Tube #1").
3. (optional) Add100µL of nuclease-free water to Tube #1.
4. Add equal volume of phenol solution (pH8; Sigma-Aldrich) to Tube #1. Mix the solution by gently inverting for 2 min and then centrifuge at 16,000 x G, 4°C, for 5min.
5. Carefully remove the top layer of the resulting 2-phase mixture with a pipette and transfer it into a clean microcentrifuge tube (Tube #2).

6. Add equal volume of the phenol: chloroform: isoamyl alcohol (25:24:1) solution (Fisher BioReagents) to Tube #2. Mix the solution by gently inverting for 2 min and then centrifuge at 16,000 x G, 4°C, for 3min.
7. Carefully remove the top layer of the resulting 2-phase mixture with a pipette and transfer it into a clean microcentrifuge tube (Tube #3).
8. Add equal volume of the chloroform/ isoamyl alcohol mixture (Sigma-Aldrich) to Tube #3. Mix the solution by gently inverting for 2 min and then centrifuge at 16,000 x G, 4°C, for 3min.
9. Carefully remove the top layer of the resulting 2-phase mixture with a pipette and transfer it into a clean microcentrifuge tube (Tube #4). Estimate the volume in Tube #4 with a pipette.
10. To Tube#4, add 1/10 of the tube volume of 3M NaAc (pH5.2). (for example, for a 300 µL volume, 30 µL NaAc was added). Follow immediately with the addition of 2 times the final volume in Tube#4 of ethanol (for example, 330 µL x 2).
11. Gently invert the tubes several times to mix the solution and place the tube at -20°C for at least 20 min.
12. Centrifuge Tube#4 at 16,000 x G, 4°C, for 10min. Remove all the supernatant carefully so as not to disturb the DNA pellet.
13. Add 500µL of ice-cold 70% ethanol mixed gently by pipetting several times. Centrifuge the tube at 5,000 x G, 4°C, for 5min.
14. Remove the supernatant completely, and let the tube air-dry for several minutes. To aid in the complete removal of excess ethanol, an aspirator can be used, so long as the DNA pellet is not disturbed.
15. Add 50 µL of nuclease-free water to the tube to re-dissolve the DNA pellet. Mix gently by pipetting up and down. Measure the DNA concentration. (NanoDrop One, Thermo Scientific)
16. The linearized DNA can be stored on ice at 4°C overnight.

<u>In vitro transcription and RNA purification</u>

1. Thaw the following reagents from the mMESSAGE mMACHINE T7 ultra transcription kit (Invitrogen):

    a. T7 2X NTP/ARCA: thaw and vortex until completely in solution
    b. T7 GTP
    c. 10X T7 reaction buffer: thaw and vortex until completely in solution
    d. nuclease-free water

    Keep items a and b on ice; leave items c and d at room temperature.

2. In a clean 1.5mL microcentrifuge tubes, assemble the reagents for the capped in vitro transcription reaction in the following order (volumes can be scaled up or down accordingly):

	Volumes (µL)
a. nuclease-free water	(180-144-*x*)
b. T7 2X NTP/ARCA	90
c. T7 GTP	18
d. 10X T7 reaction buffer	18
e. 7.2 µg linearized replicon DNA	*x*
f. T7 Enzyme Mix	18
Total reaction volume:	180

3. Mix the reaction mixture by flicking the tube gently and then briefly centrifuge to collect all the liquids to the bottom. Incubate the tube at 37°C for 2.5 hours.
4. After 2.5 hours, add 9uL TURBO DNAse to the reaction. Mix the tube again gently and continue incubating the tube at 37°C for another 15 min.
5. Purify the freshly transcribed RNA from the reaction using the Monarch Kits for RNA Cleanup (NEB) according to the manufacturer's protocol.
6. For every 180 µL reaction, add 360 µL Binding Buffer and 540 µL ethanol. Mix by pipetting gently.
7. Load 500 µL (about half) of the sample onto the column provided by the kit, inserted into a collection tube. Centrifuge the tube at 16,000 x G at room temperature for 1 min. Discard the flow-through.
8. Repeat step (7) to load all remaining samples onto the column
9. Add 500 µL of RNA Cleanup Wash Buffer to the column. Centrifuge the tube at 16,000 x G at room temperature for 1 min. Discard flow-through. Repeat this step once more.
10. Place the column back into the empty collection tube. To remove all residual solvents and buffers, centrifuge the tube once more at 16,000 x G at room temperature for 1 min.
11. Transfer the column to a clean RNAse-free 1.5mL microcentrifuge tube. For the first elution, add 100 µL nuclease-free water to the column, let sit for 5 min at room temperature. Centrifuge the tube at 16,000 x G for 1 min.
12. Place the column into a new 1.5mL microcentrifuge tube for a second elution. Add 50 µL nuclease-free water to the column, let sit for 5 min at room temperature, and centrifuge the tube at 16,000 x G for 1 min.
13. Put the eluted RNA samples on ice and measure the RNA concentrations (NanoDrop One, Thermo Fisher Scientific).

Replicon RNA electroporation

1. Seed cells in DMEM complete media (10% FBS + pen/strep) 1-2 days prior to transfection and allow to grow until 70-90% confluency.
2. To prepare the cells for electroporation, detach cells using TrypLE Select (Gibco) and wash the cells twice in PBS or DPBS without calcium or magnesium (Gibco). Detailed procedures are as follow:
    a. Aspirate media and gently rinse the cells with 10mL of PBS or DPBS (no Ca, Mg) per T-175 flask. After removing the PBS/DPBS, add 3-4mL of TrypLE Select to each flask and let incubate for 3-5min to detach cells.
    b. Add 10mL of DMEM complete media to each flask. Gently mix the cells by pipetting. Pool cells from all flasks into 50-mL Falcon tubes and centrifuge at 200 x G for 5 min. *optional: count cells prior to centrifugation.
    c. After removing supernatant, wash cells with 30mL of PBS/DPBS (no Ca and Mg), mixing gently by pipetting up and down. If more than one tube was spun, pool the cells into one tube at this step. Spin cells at 200 x G for 5 min.
    d. Repeat step (c) to wash the cells again in 30mL PBS/DPBS (no Ca and Mg).

3. After the spin from the 2nd wash, resuspend the cells in PBS/DPBS (no Ca and Mg). The volume of resuspension will depend on the estimated number of cells: i.e. For 5 x T-175 flasks of cells, resuspend cells in 50mL PBS. For 3 x T-175 flasks of cells, resuspend in 30mL PBS. Count cells.
4. Calculate the number of cells needed for electroporation. This will depend on the number of compound plates, cell density at plating, and include appropriate overage volumes.

5. Determine the amount of RNA needed for electroporation. For this work, we used 1µg RNA for every 1x 10^6 cells.
6. Determine the volume of electroporation needed to resuspend the cells. The final concentration of cells required for electroporation is 100 x 10^6 cells per mL.
7. Aliquot the appropriate volume of cells into a Falcon tube and centrifuge at 200 x G for 5min. Aspirate the supernatant.
8. Resuspend the cells in the Maxcyte electroporation buffer by gently pipetting up and down. Add the appropriate volume of RNA.
9. Transfer the cells and RNA mix into an appropriate Maxcyte Processing Assembly (PA) cuvette- different sized cuvettes are needed depending on the volume of cells/RNA mix.
10. Electroporate the cells on the Maxcyte STx, using the protocol for the appropriate cell type. After electroporation, remove the PA from the Maxcyte STx and allow it to sit at room temperature for 20 min for recovery.
14. After 20 min, transfer the cells from the PA into a 50-mL Falcon tube that contains 20-30 mL of DMEM complete media. Count the electroporated cells.
15. Adjust the cell density to the desired density for plating onto 384-well assay plates. For this work, we used 0.4 x 10^6 cells per mL, plating 10,000 cells per 50 µL well.
16. Plate the cells into compound plates using the Agilent Bravo (automated liquid dispenser fitted with a 384-well head).
17. Incubate the cells at 37°C/ 5% CO_2/ 90% relative humidity.

Compound plate preparation

1. Prepare compound stocks in DMSO. Dispense 0.2 µL/well into 384-well poly-D-lysine-coated plates (Corning 356663) or cell culture-treated, flat-bottomed plates (Corning 3571) using an ECHO acoustic dispenser. Omit the outer edges of the 384-well plate to avoid inconsistencies in plate coating.
2. For each compound, dispense a 10-point series that consists of 3-fold serial dilutions, with final concentrations ranging from 42016 nM to 2.1nM.
3. Use DMSO as the minimum inhibition control (final concentration 10 µM).

Data Collection and Cytotoxicity Assay

1. At 30 h post-plating, quantify the reporter activities in the compound-treated cells by
    a. Counting the number of green cells in each well using an Acumen eX3 scanner, or
    b. Measuring the luciferase activity with the Steady-Glo Luciferase assay system (Promega) using the EnVision (Perkin Elmer)
2. Compound toxicities in treated cells can be evaluated using a CellTiter-Glo (CTG) luminescent assay (Promega #G7571). After scanning plates on the Acumen eX3 for GFP signal, let the plates equilibrate to room temperature (20-30 min).
3. Thaw CTG reagents and let equilibrate to room temperature.
4. Following the manufacturer’s protocol, mix the reagents well before adding them to the plate.
5. Using the Agilent Bravo (fitted with a 384-well head), dispense 30µL into each well, briefly mix 2 times by pipetting up and down and let incubate for 10 min.
6. After 10 min, measure the luminescent signals using the EnVision (Perkin Elmer).
